# Neighbour presence, not identity, influences root and shoot allocation in pea

**DOI:** 10.1371/journal.pone.0173758

**Published:** 2017-03-14

**Authors:** Cory. E. Jacob, Eric Tozzi, Christian J. Willenborg

**Affiliations:** Department of Plant Sciences, University of Saskatchewan, Saskatoon, SK, Canada; Helmholtz Centre for Environmental Research - UFZ, GERMANY

## Abstract

Competition is a key feature that structures the composition of plant communities. A growing body of evidence is showing that the presence of neighbours, especially belowground neighbours, induces varied morphological responses in plants. However, in many species, it is not known whether neighbour identity also influences plant morphological responses such as biomass allocation patterns. To assess plant response to above- and belowground neighbour presence and identity, we conducted a greenhouse experiment consisting of conspecific (pea; *Pisum sativum* L.) and heterospecific (oat; *Avena sativa* L.) neighbours growing with a *P*. *sativum* focal plant. Four interaction regimes were constructed including shoot, root, or ‘full’ interaction (root & shoot) treatments, as well as a control with no interactions permitted. Our results showed that pea plants responded negatively to the presence of neighbours, and in particular, the presence of belowground neighbours. Treatments where belowground interactions were permitted (full and root interactions) had lower root and shoot mass fractions (R:S ratios) than where shoot interactions were permitted. Shoot and root allocation and R:S ratios of focal pea plants were not affected by neighbour identity, suggesting that neighbour presence, but not identity, influenced allocation patterns. The impact on *P*. *sativum* of a neighbouring competitor was more prominent than neighbour identity, showing that some plants may not discriminate between the identity of neighbours even though they are capable of responding to their presence.

## Introduction

Organisms of varying complexity are now widely recognized to differentially perceive and even respond to the identity of neighbours, including the ability to differentiate between heterospecific and conspecific neighbours. Although this type of neighbour perception is widely recognized in other taxa, it has largely been neglected in studies of plants [1,2]. It is generally accepted that plants respond to and recognize multiple biotic and abiotic stimuli, but the ability of an individual plant to recognize and respond to neighbouring plants remains a subject of much attention in the literature [3,4]. A growing body of evidence is showing that the presence of neighbours, especially belowground neighbours, induces varied morphological and chemical responses in plants [5,6]. Such responses include changes in morphological and physiological traits [7,8], root placement [9], and biomass allocation [7,10], as well as altered gene expression [11,12]. Collectively, these responses enable plants to discriminate between themselves and other plants [13,14], even between kin and non-kin [3,15].

The ability of plants to recognize and respond to all aspects of their environment will influence their competitive ability and thus, be critical to the success of a species [1]. In fact, competition is thought to be a major factor structuring weed communities, and may help to explain weed species density and community diversity [16]. Competition between heterospecifics can be markedly different than competition between conspecific plant neighbours [17], and plant neighbour identity can alter morphological traits [4,18] and gene expression [11]. In that regard, examining above- and belowground plant responses to heterospecific and conspecific neighbours is vital to understanding plant-plant interactions during competition.

The majority of plant competition takes place belowground [19], and root competition is thought to be important to crop-weed interactions [20]. Although research on belowground competition has traditionally been limited due to the difficulty in observing root interactions, the subterranean aspects of plant life have received significant attention over the past two decades [21]. It is now known that belowground traits important to competitive ability include root size, volume [22], distribution, and rate of resource uptake [23]. More impressively, plants can integrate information about nutrients and neighbours and adjust allometric distributions in response [9, 24, 25]. However, others contend that neighbour-induced root responses tend to be species-specific and environment dependent [26]. Better understanding belowground interactions between plants has relevance to the development of more competitive plants and crop varieties [27, 28, 29].

Competition that occurs aboveground is also important [9, 30], and traits such as plant height [31,32] and leaf area [33, 34] are key components of competitive crop stands. An interesting exception to this is field pea (*P*. *sativum*), wherein semi-leafless varieties that lack true leaves exhibit little overall variation in competitive traits such as plant height, but differ in competitive ability [35]. This suggests interactions belowground may play an important role in the competitive ability of this species, although it is not known whether neighbour identity influences *P*. *sativum* root and shoot allocation. Little is also known about the importance of above- versus belowground interactions in many legume species, but studies have shown that the presence of belowground neighbours can result in root overproliferation at the expense of seed production in legume species such as *Phaseolus vulgaris* L. [36] and *P*. *sativum* [37]. We tested if an individual *P*. *sativum* plant would alter its growth and allocation due to the presence of conspecific versus heterospecific plant neighbours. Here, we present the results of a greenhouse study where we manipulated plant neighbour identity (conspecific: *P*. *sativum* or heterospecific: *Avena sativa* L.) under various interaction regimes (full interaction, no interaction, shoot interaction, root interaction) to determine the relative influence of these factors on *P*. *sativum* growth and allocation.

## Materials and methods

For this study, we used pea (*P*. *sativum*) as the experimental (focal) plant because it has been shown to exhibit risk sensitivity (adjustment of rooting strategies based on risk aversion related to poor growth conditions) as an adaptive strategy [38], and responses to neighbouring roots have been reported (e.g. [7,39]). Moreover, aboveground traits are not responsible for competitive differences between varieties [35]. As a range of interaction treatments is crucial to our objective, treatment factors consisted of factorial combinations of two different neighbour species and four interaction regimes. Focal pea plants (cv. CDC Meadow) were grown with neighbours that were either conspecific *P*. *sativum* (cv. CDC Dakota) or heterospecific *Avena sativa* L. (cv. CDC Haymaker) plants. CDC Meadow was chosen because it was a commonly grown pea variety at the time of trial initiation, while CDC Dakota was shown to be one of the most competitive pea varieties [35]. Each neighbour identity treatment was grown under one of four interaction regimes consisting of full interaction, shoot interaction, root interaction, and no interaction.

**Fig 1:**
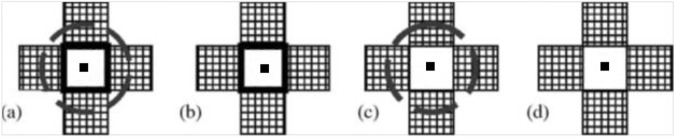
Layout of treatments for greenhouse study. Centre square represents focal plant species (pea) and grid pattern represents neighbour treatments (*P*. *sativum* or *A*. *sativa*). The solid square line represents the belowground barrier (black line in square shape around focal pea plant) while the dashed circle represents the aboveground barrier. a) No interaction–above—and belowground barriers present b) Shoot interaction–only belowground barriers present c) Root interaction–only aboveground barriers present d) Full interaction (root & shoot)–no barriers present. Adapted from Walker and King [40].

Experimental units consisted of five pots arranged in a ‘+’ shape, each with a single plant per pot (Fig 1). A single focal plant was planted in the center pot, with the four surrounding pots sown with either conspecific or heterospecific neighbours. For interaction regimes requiring belowground separation (i.e. shoot interaction and no interaction), plants remained in their individual pots with no root interaction permitted among pots [40]. When belowground separation was not needed (i.e root interaction and full interaction), the sides of the pots that were shared with another pot were removed and placed together in the ‘+’ shape. In these treatments, focal plants thus experienced very similar belowground interactions and differed only in terms of aboveground interactions. Where aboveground interaction was required, plants were allowed to interact above the soil surface (i.e. shoot interaction and full interaction), but where aboveground separation was required, plants were separated using wire mesh (aboveground barrier) installed 10 d after planting. In these treatments, focal plants thus experienced very similar aboveground interactions and differed only in terms of belowground interactions. Aboveground barriers, intended to minimize interactions, were made of 24 gauge galvanized welded iron mesh with 6mm openings. Wire mesh was cut into 60 cm by 52.5 cm pieces, and folded into a freestanding tube that fit into each pot. The aboveground barriers generally intercepted less than 20% of the available photosynthetically active radiation (PAR). Each experimental unit, consisting of five pots, was re-randomized weekly to minimize environmental variability.

A 3:1 mixture of sand:soil (dark brown chernozemic soils) was utilized as the potting medium. The mixture was thoroughly mixed together and watered to field capacity before potting. Seeds were sown in 13 cm diameter (2 L) pots at a depth of 5 cm. All seeds were pre-germinated for 2 d before planting to ensure uniform germination and emergence. *P*. *sativum* seeds were inoculated with the appropriate strain of rhizobium species (*Rhizobium leguminosarum* biovar viceae) at a rate of 0.2% w/w prior to planting. A square pot-planter 13 cm wide, was constructed to ensure uniform planting depth, and that plants were equidistant and equiangular to each other to eliminate any competitive bias [41]. Neighbouring plants were spaced 13 cm from the focal plant.

A two-factor, randomized complete block design with four replicates was utilized. Greenhouse temperatures in both experimental runs were maintained at 24/20°C day/night with an 18-h photoperiod. Artificial lighting was provided by 1000-W high-pressure sodium lamps with a photosynthetically active radiation (PAR) level below 1000 μmol m^-2^ s^-1^, and were turned off when PAR was above 1300 μmol m^-2^ s^-1^. Relative humidity was 38% and 59% for the first and second experimental runs, respectively. Plants were monitored daily and were watered to field capacity as necessary. Each plant received a nutrient dose of 20-20-20 fertilizer (100 μg ml^-1^ solution) at a rate of 11 kg ha^-1^ twice throughout each experimental run (bi-weekly).

Plants were harvested just prior to the focal plant flowering. At this time, vine length was measured for the focal plants from the soil surface to the top of the apical meristem, and leaf area was determined by cutting all of the leaves off each focal plant and passing them through a leaf area meter. Aboveground biomass (shoot) was taken for the focal plants by cutting the plants at the soil surface, placing them in paper bags, drying them at 40°C for 48 h, and then weighing them. Root biomass was measured by very carefully removing any adhering sand from the roots, soaking the roots in water for 3–5 min, and then carefully separating the roots of each plant. Once separated, roots were placed into paper bags, dried for 48 h at 40°C, and were then weighed. Subsequently, total mass, root mass fraction and shoot mass fraction were determined to calculate root:shoot (R:S) ratios.

## Statistical analysis

Linear mixed models were constructed using the MIXED model procedure of SAS [42], with vine length, leaf area, root and shoot biomass, and root:shoot ratios as response variables. Residuals were initially tested for normality with the UNIVARIATE procedure, while homogeneity of error variance was confirmed using Levene’s test in SAS [42]. Root to shoot ratios were log_10_ transformed for analysis and then back-transformed for presentation. Fixed effects in the model were the four interaction regimes, neighbour species, and the regime*neighbour interaction, while the random effects consisted of block nested in experimental run and experimental run itself. The random effects were examined using COVTEST to see if experimental runs could be combined, which they could for all response variables. Means separation was performed using Tukey’s HSD at *P* < 0.05.

## Results

**Fig 2:**
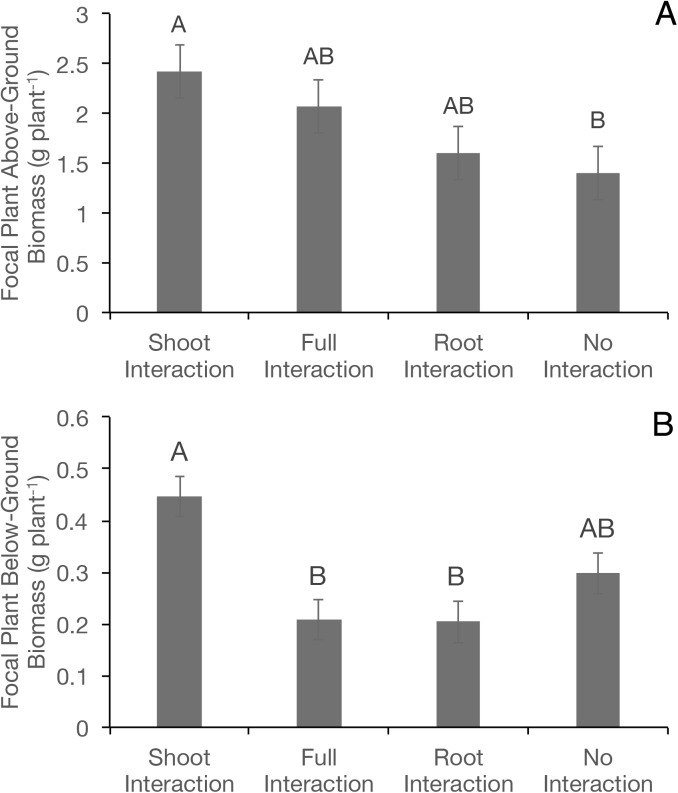
**Focal plant shoot (A) and root (B) biomass among various interaction regimes in a greenhouse experiment.** Error bars represent the standard error of the least squares means. Similar letters indicate no significant difference at HSD_0.05._

Focal pea plants grown with conspecific or heterospecific plant neighbours produced similar vine lengths and leaf area, regardless of whether root or shoot interactions were permitted (Table 1). On the other hand, focal plant shoot biomass differed (*P* < 0.05) among interaction regimes, but not among neighbour identities (Table 1). Shoot biomass in focal plants was 73% greater when only shoot interaction was permitted compared with the no interaction treatment (Fig 2). Full and root interaction treatments were intermediate, and did not differ from either the shoot or the no interaction treatments. No statistically significant interaction between neighbour identity and interaction regime was detected for focal pea shoot biomass (Table 1).

**Table 1:** ANOVA table for focal (pea) plant vine length (VL), leaf area (LA), focal shoot biomass (SBM), root biomass (RBM), and root:shoot ratio (R:S) in a greenhouse experiment.

		VL		LA		SBM		RBM		R:S	
	DF	F-value	P-value	F-value	P-value	F-value	P-value	F-value	P-value	F-value	P-value
Neighbour (N)	1,30	0.267	0.585	0.729	0.624	0.153	0.908	0.604	0.438	0.315	0.602
Interaction Regime (IR)	3,30	1.155	0.301	2.600	0.281	5.359	0.039*	9.596	0.001***	6.142	0.001***
N X IR	3,30	1.244	0.381	0.684	0.808	0.769	0.760	0.730	0.675	0.298	0.727
	DF	Z-value	P-value	Z-value	P-value	Z-value	P-value	Z-value	P-value	Z-value	P-value
Run	1,30	0.310	0.614	2.890	0.182	1.336	0.205	0.685	0.445	0.562	0.239
Rep	3,30	0.612	0.477	2.612	0.112	1.113	0.188	1.023	0.193	0.324	0.308

*, **, ***, significant at the 0.05, 0.01, and 0.001 probability levels

Focal plant root biomass also differed among interaction treatments, but not among neighbour identities (Table 1). The presence of a belowground neighbour (full or root interaction regimes) had significant (*P* < 0.001) negative effects on focal plant root biomass, as root biomass of the focal plant was over two-fold lower in the presence of a belowground neighbour compared with an aboveground neighbour (Fig 2). Root biomass of the focal plant in the presence of a belowground neighbour did not differ from either the no or the full interaction treatments, which suggests that the focal plant increased the production of root biomass in response to the presence of aboveground interactions from the neighbouring species.

**Fig 3:**
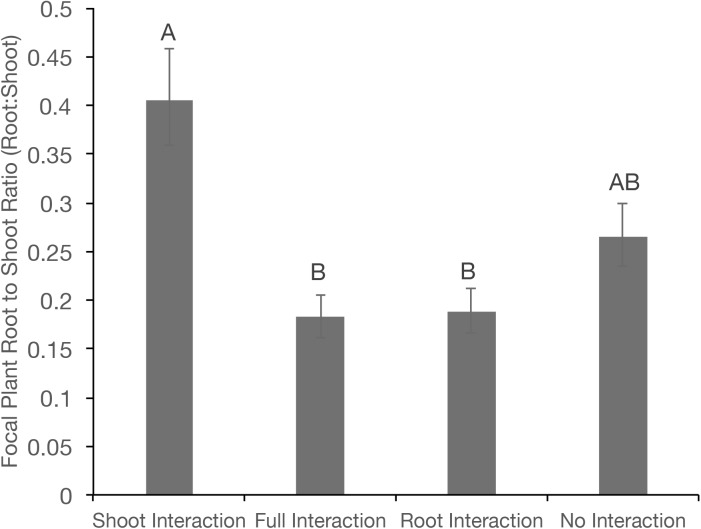
Focal plant root:shoot (R:S) ratio among various interaction regimes in a greenhouse experiment. Error bars represent the standard error of the least squares means. Similar letters indicate no significant difference at HSD_0.05._

Root:shoot ratio (R:S) of focal pea plants was not affected by neighbour identity, nor was there a statistical interaction between neighbour identity and interaction regime (Table 1). However, differences in allocation patterns were reflected in focal plant R:S ratios, which differed between interaction regimes, but not between neighbour identities (Table 1). The largest R:S ratio was observed in treatments that permitted shoot interaction (0.41), although this did not differ from the no interaction treatment (Table 1; Fig 3). Treatments that permitted belowground interactions (full or root interaction), on the other hand, had lower R:S ratios (*P*< 0.001 for shoot interaction) than the other treatments where shoot interactions were permitted; R:S ratios were more than two-fold greater for treatments with shoot interactions compared with root interaction or full interaction treatments (Fig 3).

## Discussion

By setting up an experiment wherein pea plants were permitted to interact with neighbours in various ways, we determined that above- and belowground interactions influenced the relative allocation to different tissues in focal pea plants. Moreover, the results showed that pea plants responded to the presence of neighbours, and in particular, the presence of belowground neighbours. Root production tended to be more adversely affected by neighbours than shoot production; pea plants allocated less mass to roots when exposed to belowground neighbours, regardless of neighbour identity. Both root mass and R:S ratio declined substantially in focal pea plants when belowground neighbours were present compared with treatments in which belowground neighbours were absent. It is possible this was due to differences in rooting volume between the treatments, as plants without root barriers theoretically had access to five times the soil volume of plants with root barriers. Rooting volume has been shown to have an impact on the outcome of neighbour interactions in other studies [6, 43]. This effect was not caused by nutrient toxicity, however, because we kept nutrient amounts per plant constant. It also was not caused by water limitation because we supplied sufficient amounts of water throughout the experiment, and water was provided at a consistent rate across all treatments. Moreover, had drought stress occurred, it would likely have led to an increased root mass, while we observed a reduction in this trait.

A possible explanation for the observed reduction in root traits when peas interacted with neighbours belowground might be that the presence of belowground neighbours enhances root respiration and increases root secretions [6]. Meier et al. [7] observed a 29% increase in oxygen consumption in pea roots grown in the presence of neighbours, which would lead to lower root mass in plants with neighbours. Another possibility, however, is that the reductions in root traits that we observed may be the by-product of root avoidance strategies to avoid belowground neighbours. Indeed, shoot interaction treatments in our case prompted greater root biomass accumulation, but root biomass was significantly reduced with removal of the belowground barriers, which may be indicative of an avoidance strategy by the focal pea plant. Numerous studies have documented that plants, including pea, are able to restrict root growth towards the direction of the neighbouring plant, thereby preferentially allocating roots away from that neighbour [9, 43, 44]. The extent to which this response may affect total plant mass remains unclear, as we did not attempt to isolate this phenomenon and its potential contribution to shoot and root mass fractions. However, a recent study has successfully linked plasticity in R:S ratios with competitive effects [25]. A third possibility is that these species may exude allelopathic substances that are autotoxic to the growth of neighbours, thereby reducing root and shoot growth of neighbouring plants. Recent studies have shown that the root exudates of both *P*. *sativum* [45] and *A*. *sativa* [46] can inhibit the growth of con- and/or heterospecific neighbours.

Our finding that peas grown with belowground neighbours had less total mass (lower root and shoot mass) does suggest that belowground neighbour presence can affect plant growth, regardless of neighbour identity. Surprisingly, pea plants in our study allocated less mass to roots when exposed to belowground neighbours, which is in agreement with Chen et al. [6]. However, this is opposite to the root–overproliferation scenario predicted by the tragedy of the commons hypothesis, which also has been observed in pea [37]. It is possible that pea exhibits species-specific or even genotypic-specific responses to neighbour presence, although this has yet to be tested. It is also possible that in studies that frequently flush the soil substrates, such as O’Brien et al. [37], autotoxic belowground signals may be leached out, producing weakened negative effects of neighbour presence [6]. Nevertheless, our data demonstrate the importance of belowground interactions in determining pea growth, and suggest that belowground interactions are likely to determine the outcome of competition in pea. The large reduction in focal plant root and shoot biomass associated with root interactions lends support to the notion that root interactions are of greater importance than shoot interactions, as concluded by Casper and Jackson [19]. Ultimately, intense root interactions still may prove unimportant for community structure because of the size-asymmetry of competition [47].

Our results show that neighbouring plants had strong effects on plant growth, even though all treatments were grown under homogenous conditions with a consistent, non-limiting supply of water and nutrients. Interestingly, we observed greater aboveground and belowground biomass when shoot interactions were permitted, which suggests that shoot interaction can stimulate the growth of shoots and especially roots. This may be a shade avoidance response to neighbours, wherein focal *P*. *sativum* plants detected to the presence of aboveground neighbours via changes in light quality, or the ratio of red to far-red light, R:FR [48]. Aboveground detection of neighbouring plants has been shown to reduce *Glycine max* (L.) root length, surface area, and volume [49], while increasing early season internode elongation, and plant height [50]. Alternatively, the presence of aboveground neighbours could have facilitated greater climbing by *P*. *sativum* plants, which may have led to increased shoot biomass due to improved light interception.

Biomass allocation patterns shifted when neighbours were allowed to interact either aboveground or belowground, which resulted in variations of R:S ratios for these treatments. We observed higher R:S ratios when neighbours only interacted aboveground compared with treatments which also permitted belowground interactions. This is interesting as light competition typically results in increased allocation to shoots at the expense of roots, producing a lower R:S ratio [51, 52]. It is plausible that there was simply no response in these aboveground traits to the presence of neighbours. Alternatively, competition for light may have been negligible in our study, or if present, had a very limited effect on plant growth because R:S ratios in the presence of aboveground competitors did not differ from treatments that permitted no interactions with neighbours. Armas and Pugnaire [53] also failed to observe changes in R:S ratios in plants grown with neighbours compared to plants grown without neighbours.

Determining if plants detect and respond to neighbours is critical to better understand plant interactions. While it is generally accepted that plant interactions between heteropecifics can differ vastly from interactions between conspecific neighbours [17], the importance of identity of a competitor remains largely unresolved. Researchers have documented significant effects of neighbour genotype on the performance of focal plants [53,54], while others failed to observe such a response [55]. In our case, pea plants did not respond to the identity of neighbours. *P*. *sativum* plants with heterospecific neighbours exhibited similar resource allocation patterns to plants with conspecific neighbours, regardless of whether interactions occurred above or below the soil surface. This suggests that effects of interacting with a neighbour are more prominent than the effects of neighbour identity in the presence of only a single neighbour. Our finding that pea plants did not alter their pattern of biomass allocation in response to neighbour identity concurs with the findings of Cahill et al. [56], and lends support to the hypothesis that competition does not always cause niche differentiation in plants [56,57]. Two possible reasons for this are that niches for the species used in our study (*P*. *sativum* and *A*. *sativa*) may be separated temporally and not spatially, or niches may actually be less important than other ecological factors with regard to coexistence [58,59]. Further research is required to test these hypotheses as they pertain to the species used in this study.

Overall, the data indicate that the impact on pea plants of a neighbouring plant was more prominent than neighbour identity. This finding improves our understanding of how plants respond to neighbours, which is likely to be important to both plant breeders and ecologists. Because we used a single density of neighbouring species, our results do not permit prediction of stand-level responses under field conditions. Nevertheless, our data show that some plant species do not discriminate between the identity of neighbours even though they are capable of responding to their presence. This has important implications for crop-weed competition research, as crop plants are typically surrounded by neighboring heterospecifics (weeds) and conspecifics (crops). Recent research has documented the ability of corn (*Zea mays* L.) and soybean (*Glycine max* L.) plants to respond to the presence of neighbouring plants through shifts in light quality [60,61]. This research does not consider the impact of neighbouring conspecifics, however, and it is currently unknown whether these crop species would respond to conspecific neighbours in the same manner. It is possible that these species respond in a similar fashion to the presence of any neighbouring plant, regardless of identity, as we have observed for pea plants. Plant species differ in the way they are affected by neighbouring plants [62], and the ability of species to respond to the identity of neighbours is highly species-specific [63], with important consequences for competitive outcomes [25]. Given that we failed to observe a response to neighbour identity in *P*. *sativum*, our research provides further evidence to show that discriminatory mechanisms are not universal among plant species.

## Supporting information

S1 File Raw data used the first experimental run of the greenhouse trial (Figs 2 and 3). (XLSX)

S2 File Raw data used the second experimental run of the greenhouse trial (Figs 2 and 3). (XLSX)

## References

[1] Broz AK, Broeckling CD, De-la-Pena C, Lewis MR, Greene E, Callaway RM et al Plant neighbor identity influences plant biochemistry and physiology related to defense. BMC Plant Biology. 2010; 10:115 10.1186/1471-2229-10-115 20565801 PMC3095278

[2] Karban R. Plant behaviour and communication. Ecology Letters. 2008; 11:727–739. 10.1111/j.1461-0248.2008.01183.x 18400016

[3] Dudley SA, File AL. Kin recognition in an annual plant. Biology Letters. 2007; 3:435–438. 10.1098/rsbl.2007.0232 17567552 PMC2104794

[4] Milla RN, Forero DM, Escudero AN, Iriondo JM. Growing with siblings: a common ground for cooperation or for fiercer competition among plants? Proceedings of the Royal Society B: Biological Sciences. 2009; 276:2531–2540. 10.1098/rspb.2009.0369 19403541 PMC2686667

[5] Chen BJW, During HJ, Anten NPR. Detect thy neighbour: identity recognition at the root level in plants. Plant Science. 2012; 195:157–167. 10.1016/j.plantsci.2012.07.006 22921010

[6] Chen BJW, During HJ, Vermeulen PJ, de Kroon H, Poorter H, Anten NPR. Corrections for rooting volume and plant size reveal negative effects of neighbour presence on root allocation in pea. Functional Ecology. 2015; 29:1383–1391.

[7] Meier IC, Angert A, Falik O, Shelef O, Rachmilevitch S. Increased root oxygen uptake in pea plants responding to non-self neighbours. Planta. 2013; 238:577–586. 10.1007/s00425-013-1910-4 23779000

[8] Semchenko M, Saar S, Lepik A. Plant root exudates mediate neighbour recognition and trigger complex behavioural changes. New Phytologist. 2014; 204:631–637. 10.1111/nph.12930 25039372

[9] Cahill JF, McNickle GG, Haag JJ, Lamb EG, Nyanumba SM, St Clair CC. Plants integrate information about nutrients and neighbours. Science. 2010; 328:1657 10.1126/science.1189736 20576883

[10] Bhatt VM, Khandelwal A, Dudley SA. Kin recognition, not competitive interactions predicts root allocation in young *Cakile edentula* seeding pairs. New Phytologist. 2011; 189:1135–1142. 10.1111/j.1469-8137.2010.03548.x 21118260

[11] Horvath DP, Llewellyn D, Clay SA. Heterologous hybridization of cotton microarrays with velvetleaf (*Abutilon theophrasti*) reveals physiological responses due to corn competition. Weed Science. 2007; 55:546–557.

[12] Schmid C, Bauer S, Muller B, Bartelheimer M. Belowground neighbor perception in *Arabidopsis thaliana* studied by transcriptome analysis: roots of *Hieracium pilosella* cause biotic stress. Frontiers in Plant Science. 2013; 4:296 10.3389/fpls.2013.00296 23967000 PMC3743015

[13] Gundel PE, Pierik R, Mommer L, Ballare CL. Competing neighbors: light perception and root function. Oecologia. 2014; 176-1-10. 10.1007/s00442-014-2983-x 24894371

[14] Pierik R, Mommer L, Voesenek LACJ, Robinson D. Molecular mechanisms of plant competition: neighbour detection and response strategies. Functional Ecology. 2013; 27:841–853.

[15] Dudley SA, Murphy GP, File AL. Kin recognition and competition in plants. Functional Ecology. 2013; 27:898–906.

[16] Booth BD, Swanton CJ. Assembly theory applied to weed communities. Weed Science. 2002; 50:2–13.

[17] Fonteyn PJ, Mahall BE. Competition among desert perennials. Nature. 1978; 275:544–545.

[18] Gruntman M, Novoplansky A. Physiologically mediated self/non-self discrimination in roots. Proceedings of the National Academy of Sciences USA. 2004; 101:3863–3867. 10.1073/pnas.0306604101 PMC374335 15004281

[19] Casper BB, Jackson RB. Plant competition underground. Annual Review Ecology and Systematics. 1997; 28:545–570.

[20] Wilson JB. Shoot competition and root competition. Journal of Applied Ecology. 1988; 25: 279–296.

[21] Cahill JF, McNickle GG. The behavioural ecology of nutrient foraging by plants. Annual Review Ecology and Systematics. 2011; 42:289–311.

[22] Gaudet CL, Keddy PA. A comparative approach to predicting competitive ability from plant traits. Nature. 1988; 334:242–243.

[23] Dunbabin V. Simulating the role of rooting traits in crop-weed competition. Field Crops Research. 2007; 104:44–51.

[24] Weiner J. Allocation, plasticity, and allometry in plants. Perspectives in Plant Ecology, Evolution, and Systematics. 2004; 6:207–215.

[25] Bennett JA, Riibak K, Tamme R, Lewis RJ, Meelis P. The reciprocal relationship between competition and intraspecific trait variation. Journal of Ecology. 2016; 104:1410–1420.

[26] McNickle GG, Brown JS. An ideal free distribution explains the root production of plants that do not engage in a tragedy of the commons game. Journal of Ecology. 2014; 102:963–971.

[27] Brown DA, Scott HD. Dependence of crop growth and yield on root development and activity 1984 p. 101–136. *In*: Barber S.A., Bouldin D.R., Kral D.M., and Hawkins S.L. (eds.) Roots, nutrient and water influx, and plant growth. Soil Science Society of America Madison, Wisconsin.

[28] Mackay AD, Barber SA. Effect of nitrogen on root growth of two corn genotypes in the field. Agronomy Journal. 1986; 78: 699–703.

[29] Koscielny CB, Gulden RH. Seedling root length in *Brassica napus* L. is indicative of seed yield. Canadian Journal of Plant Science. 2012; 92:1229–1237.

[30] O’Donovan JT, Harker KN, Clayton GW, Hall LM. Wild oat (*Avena fatua*) interference in barley (*Hordeum vulgare*) is influenced by barley variety and seeding rate. Weed Technology. 2000; 14:624–629.

[31] Murphy KM, Dawson JC, Jones SS. Relationship among phenotypic growth traits, yield and weed suppression in spring wheat landraces and modern cultivars. Field Crops Research. 2008; 105:107–115.

[32] Zerner MC, Gill GS, Vandeleur RK. Effect of height on the competitive ability of wheat with oats. Agronomy Journal. 2008; 100: 1729–1734.

[33] Cote R, Gerrath M, Poslusznyu A, Grodzinski B. Comparative leaf development of conventional and semi-leafless peas (*Pisum sativum*). Canadian Journal of Botany. 1992; 70: 571–580.

[34] Radosevich S, Holt J, Ghersa C. Plant-plant associations 2007 pp 183–237 *in* Radosevich S, Holt J, Ghersa C eds. Ecology of Weeds and Invasive Plants: Relationship to Agriculture and Natural Resource Management. New Jersey: John Wiley and Sons, Inc.

[35] Jacob CE, Johnson EN, Dyck MF, Willenborg CJ. Evaluating the competitive ability or semileafless field pea cultivars. Weed Science. 2016; 64:137–145.

[36] Maina GG, Brown JS, Gersani M. Intra-plant versus inter-plant root competition in beans: avoidance, resource matching or tragedy of the commons. Plant Ecology. 2002; 160:235–247.

[37] O’Brien EE, Gersani M, Brown JS. Root proliferation and seed yield in response to spatial heterogeneity of below-ground competition. New Phytologist. 2005; 168:401–412. 10.1111/j.1469-8137.2005.01520.x 16219079

[38] Dener E, Kacelnik A, Shemesh H. Pea plants show risk sensitivity. Current Biology. 2016; 26:1763–1767. 10.1016/j.cub.2016.05.008 27374342

[39] Falik O, Reides P, Gersani M, Novoplansky A. Self/non-self discrimination in roots. Journal of Ecology. 2003; 91:525–531.

[40] Walker JA, King JR. Above- and below-ground competition between Kura clover and meadow bromegrass: A greenhouse study. Can Journal of Plant Science. 2009; 89:21–27

[41] Willenborg CJ, Rossnagel BN, Shirtliffe SJ. Oat caryopsis size and genotype effects on wild oat–oat competition. Crop Science. 2005; 45:1410–1416.

[42] SAS Institute. 2011 SAS User’s Guide. Version 9.3. Cary, NC: SAS Institute.

[43] Hess L, de Kroon H. Effects of rooting volume and nutrient availability as an alternative explanation for root/non-self discrimination. Journal of Ecology. 2007; 95:241–251.

[44] Mahall BE, Callaway RM. Root communication among desert shrubs. Proceedings of the National Academy of Sciences USA. 1991; 88:874–876. 10.1073/pnas.88.3.874 PMC50916 11607151

[45] Asaduzzaman M, Asao T. Autotoxicity in beans and their allelochemicals. Scientia Horticulturae. 2012; 134:26–31.

[46] Semchenko M, Hutchings MJ, John EA. Challenging the tragedy of the commons in root competition: confounding effects of neighbour presence and substrate volume. Journal of Ecology. 2007; 95:252–260.

[47] Lamb EG, Kembel SW, Cahill JF. Shoot, but not root, competition reduces community diversity in experimental mesocosms. Journal of Ecology. 2009; 97:155–163.

[48] Franklin KA. Shade Avoidance. New Phytologist. 2008; 179:930–944. 10.1111/j.1469-8137.2008.02507.x 18537892

[49] Gal J, Afifi M, Lee E, Lukens L, Swanton CJ. Detection of neighboring weeds alters soybean seedling roots and nodulation. Weed Science. 2015; 63:888–900.

[50] Green-Tracewicz E, Page ER, Swanton CJ. Shade avoidance in soybean reduces branching and increases plant-to-plant variability in biomass and yield per plant. Weed Science. 2011; 59:43–49.

[51] Gersani M, Abramsky Z, Falik O. Density-dependent habit selection in plants. Evolution and Ecology. 1998; 12:223–234.

[52] Murphy GP, Dudley SA. Above- and below-ground competition cues elicit independent responses. Journal of Ecology. 2007; 95:261–272.

[53] Armas C, Pugnaire FI. Plant neighbour identity matters to belowground interactions under controlled conditions. PLoS ONE. 2011; 6: e27791 10.1371/journal.pone.0027791 22114696 PMC3219686

[54] Fridley JD, Grime JP, Bilton M. Genetic identity of interacting neighbours mediates plant responses to competition and environmental variation in a species rich grassland. Journal of Ecology. 2007; 95:908–915.

[55] Lankau RA, Wheeler E, Bennett AE, Strauss SY. Plant-soil feedbacks contribute to an intransitive competitive network that promotes both genetic and species diversity. Journal of Ecology. 2001; 99:176–185.

[56] Cahill JF, Kembel SW, Gustafson DJ. Differential genetic influences on competitive effect and response in *Arabadopsis thaliana*. Journal of Ecology. 2005; 93:958–967

[57] Harper JL. Plant population biology. Academic Press 1977. 892 pp.

[58] Bazzaz F. Plants in changing environments: linking physiological, population, and community ecology. Cambridge Univ. Press 1996. 320 pp.

[59] Cheeson PL. A need for niches? Trends in Ecology and Evolution. 1991; 6:26–28. 10.1016/0169-5347(91)90144-M 21232416

[60] Afifi M, Swanton CJ. Maize seed and stem roots differ in response to neighbouring weeds. Weed Research. 2011; 51:442–450.

[61] Mckenzie-Gopsill AG, Lee E, Lukens L, Swanton CJ. Rapid and early changes in morphology and gene expression in soya bean seedlings emerging in the presence of neighbouring weeds. Weed Research. 2016; 56:267–273.

[62] Pierik R, Mommer L, Voesenek LACJ. Molecular mechanisms of plant competition: neighbour detection and response strategies. Functional Ecology. 2013; 27:841–853.

[63] Wardle DA, Peltzer DA. Interspecific interactions and biomass allocation among grassland plant species. Oikos. 2003; 100:497–506.

